# Can the Red-Green Duochrome Test Be Used Prior to Correcting the Refractive Cylinder Component?

**DOI:** 10.1371/journal.pone.0118874

**Published:** 2015-03-16

**Authors:** Liat Gantz, Shlomo Schrader, Ruthie Ruben, Ari Z. Zivotofsky

**Affiliations:** 1 Department of Optometry and Vision Science, Hadassah Academic College, Jerusalem, Israel; 2 Department of Optometry, Bar Ilan University, Ramat Gan, Israel; 3 The Leslie and Susan Gonda (Goldschmied) Multidisciplinary Brain Research Center, Bar-Ilan University, Ramat Gan, Israel; 4 Brooklyn College, City University of New York, New York, New York, United States of America; State University of New York Downstate Medical Center, UNITED STATES

## Abstract

**Purpose:**

A primary task of the eye care professional is determining the refraction, or optical correction, of a patient. The duochrome red-green test is a standard tool for verification of the final refraction. Traditionally, it is recommended for use both prior to and subsequent to determining the cylindrical or astigmatic component of the refraction. In order for it to be effective when used before correcting the cylinder it is necessary that the COLC (Circle of Least Confusion) be on the retina. This study examined whether it is necessarily true that the duochrome response in uncorrected astigmatism will be as trust-worthy as it is with corrected cylinders.

**Methods:**

The red-green examination was performed monocularly under the following three conditions: a. fully corrected refraction for the subgroup of eyes that had spherical refractions and for the subgroup of eyes with sphero-cylindrical refractions. b. best sphere-only correction without cylinder correction in sphero-cylindrical eyes c. an induced cylinder error in spherical eyes. The interval between the last “red” response and the first “green” response for the right eyes as a group and separately for the physiological cylinder and induced cylinder correction sub-groups was calculated and compared using a paired, two-tailed t-test.

**Results:**

The intervals between “red” and “green” responses were not significantly different in the population as a whole and in the uncorrected physiological cylinder and induced cylinder subgroups examined.

**Conclusion:**

Based on the finding that the interval of red-green equality with fully corrected cylinder and without the cylindrical correction are not significantly different, the red-green duochrome test can indeed be used both before and after cylindrical correction.

## Introduction

A primary function of eye care is determining the refraction of a patient, i.e. the degree of optical correction required to give the patient their best possible vision, a task that can be time consuming and relies on patient cooperation. For over 60 years textbooks have suggested that the duochrome test can be used clinically for verification of the final refraction [1]. The red-green duochrome test is also often used in research protocols to prevent over or under correction for distance [2–3], as a final fine-tuning of the spherical component [4], and to provide a final refraction [5] when a discrepancy exists between other methods [6]. Since its introduction by Brown in 1927, the red-green duochrome test has become a popular clinical tool [7].

Unlike a spherical optical system which focuses a single source object into a singular point on the retina, an astigmatic optical system (such as the eye) does not focus light into a single point. Rather, due to the unequal refracting powers of the various planes or meridians of the eye’s optical system, a single source object focuses at two varying locations (hence “a-stigma-tism”—lacking a point focus) differing in their distance from the retina. These foci represent the maximum focal power and the minimum focal power of the system, a result of the varied power of the planes of the eys’e optical power. The distance between these two focal power points is often called the Interval of Sturm. In the eye, when these two focal points are equidistant from the retina, the resulting blurred image from a point source object assumes the shape of a circle. This circle is known as the Circle of Least Confusion, and is regarded as rendering the least blurred (hence the word “confusion”) image attainable.

The Duochrome Test is based on the principle of axial chromatic aberration, that is, that the shorter wavelengths of light (i.e., green) is refracted more by the eye’s optics than longer wavelengths of light (i.e., red). The duochrome test examines the position of the focus of the green and red wavelengths with respect to the retina and theoretically enables precise determination of the spherical correction or of the Circle of Least Confusion (COLC).

The duochrome test involves the projection of black letters or symbols onto a bipartite green (at approximately 535nm) and red (at approximately 620 nm) background [8]. The red and green wavelengths are dioptrically equidistant, approximately 0.25 D, from the yellow wavelength (570 nm). The green wavelength focuses in front of the retina and the red wavelength behind the retina. A diopter (D) is the inverse of a focal length and is the standard unit of optical correction. It is assumed that best vision is attained when the yellow wavelengths are focused on the retina. Therefore, an eye that is properly corrected to be emmetropic, where light from infinity focuses on the retina, should view the black letters or symbols on both the green and red backgrounds with equal clarity. If the focus is anterior to the retina, as in the case of myopia, the letters will be blurred more for the green side, as the green focus is anterior to the yellow focus. This will indicate an overplussed or underminused dioptric refraction. If the focus is posterior to the retina, as in the case of hyperopia, the letters will be blurred more on the red side, indicating an underplussed or overminused refraction.

If a +0.25 D spherical lens is placed before the emmetropic eye, wavelengths are shifted towards the front of the eye by 0.25 D, and the patient is expected to report that the letters on the red background appear clearer [9]. Based on the same principle, if a -0.25 D spherical lens is placed before the eye, the focus of all the wavelengths is shifted posteriorly, and the patient is expected to report that the letters on the green background appear clearer. Nonetheless, Sivak [10] reports that a correction based on the duochrome (or, as he calls it, bichrome) test will produce a slight overcorrection (too much minus) for myopia and undercorrection (insufficient plus) for hyperopia, usually on the order of 0.25 D.

The Red-Green duocohrome test is traditionally recommended for use both prior to determining the cylindrical component of the refraction using a Jackson Cross Cylinder (JCC) and subsequent to this determination [8, 11]. The Jackson Cross Cylinder (JCC) is a lens comprised of a pair of cylinders of equal power, one convex and one concave, with their axes 90 degrees to each other [12]. As a result, placing the lens in front of a perfect optical system, separates the image into two focal points, one anterior, and one posterior, and equidistant from the retina. The JCC thus creates an astigmatic interval, whose blur circle is round, and whose dioptric value is the cylindrical component of the lens itself. In one JCC position, the astigmatic interval collapses and vision improves. In the perpendicular position, the astigmatic interval expands and vision deteriorates. When the image quality improves and the JCC is at the previously determined correcting axis, additional correcting cylinder is indicated. When the image quality deteriorates and the JCC is at the previously determined correcting axis, less cylinder is indicated. For example, if the previously determined correcting axis is 90, and the JCC is placed with its minus cylinder axis at 90, an improvement in vision indicates additional cylinder power. If in this JCC position vision deteriorates, cylinder power should be reduced.

When using the duochrome test before correcting cylinder with the JCC, the underlying assumption is that the COLC is on the retina. Otherwise the use of the JCC will not allow for the placement of the final cylindrical component on the retina to provide optimal visual correction. The explanation of this assumption is thus: The JCC for power correction creates two possibilities; one in which the principal meridians collapse towards each other (axis of JCC parallel to the axis of assumed correcting cylinder), and the other when the principal meridians distance themselves from each other (axis of JCC perpendicular to the axis of assumed correcting cylinder). When the COLC is on the retina and the meridians collapse towards each other, each one approaches the retina and visual acuity is improved. In the other mode, each meridian is further from the retina, and visual acuity is reduced. When the COLC is not on the retina, if, for example, it is myopic, the collapsing of the interval will not necessarily improve acuity. As a result, any correction will be myopic. In the other mode, the distancing of the meridians from each other will not necessarily reduce acuity, as one meridian may actually be focused on the retina, even though the other is distanced from it. Hence, the benefit that the JCC endows, that of presenting two visual options, one that improves acuity and one that decreases acuity, is lost, thus invalidating the test.

The position of the interval of Sturm, and COLC may also vary with the state of accommodation. Loo and Jacobs [13] reported that the accuracy of the spherical refraction did not produce any significant change in the precision of JCC axis determination, irrespective of whether the patient had up to 0.75D of myopia or hyperopia. However, the present study addressed the effectiveness of the duochrome in preparation for JCC power determination, which is a separate experimental question. As described by Borish [11] (pp. 745–746), when determining the power of the cylinder, it is necessary for the COLC to be on the retina. This experiment tested the assumption that the duochrome test places the COLC on the retina. The possible theoretical problem with the duochrome in achieving the placement of the COLC on the retina, is that the major and minor meridians each have their own unique duochrome position and may induce unreliable or even contradictory responses as to whether the green side or the red side is clearer. As such, the current study addressed the question of whether the patient necessarily responds to the average of these two chromatic aberrations, i.e. the COLC, or arbitrarily chooses the chromatic aberration of one of the meridians while ignoring the other. If the latter possibility had been found, it would have resulted in either a wider range of equality, or unreliable responses (the patient switching responses from one meridian to the other during the test itself). In other words, the study may be rephrased as: “Is the duochrome test as trust-worthy in uncorrected cylinder as it is in corrected cylinders”?

This question is highly relevant because many practitioners especially, but not only, in Europe rely heavily on the duochrome test to determine the position of the COLC. Kurtz & Carlson [14], a standard textbook used in the UK and the basis for the Israeli Optometric Association Board certification examination, states regarding the duochrome test: “is used to determine the correcting spherical lens power.” and “should be used as the endpoint procedure for the initial maximum plus to maximum visual acuity (MPMVA—defined below).” The word “initial” in this context is the use of the duochrome before introducing the JCC for cylinder verification. The diagram-flow chart in the book clearly shows the duochrome prior to the JCC. Similarly, in the US, the preparatory book for the Optometry Boards by Wilkinson & Doan [15], describes the duochrome test as performed prior to the JCC.

In other words, after determining the objective refractive status of the eye using autorefractometry or retinoscopy, the practitioner initiates a monocular subjective examination. The practitioner adds fogging plus lenses (or reduces minus lenses) to induce a myopic blur, commonly known as fogging, and then gradually removes the fogging lenses until optimal visual acuity is reached- the maximum plus to maximum visual acuity—MPMVA. Although there is no research literature that indicates that any best vision sphere procedure is better than another, the MPMVA technique of bringing the patient out of myopic blur has the advantage that accommodation is well controlled when examining young patients and is relatively easy to use with a phoropter [16], The phoropter is a standard instrument for objective and subjective refraction determination, as well as the primary tool for measuring binocular function. The duochrome test is used at this point to verify that the COLC is on the retina. Immediately following this, the JCC is introduced and the cylinder power and axis are determined.

To reiterate, it is generally agreed that towards the end of the subjective examination, after astigmatism has been corrected, the duochrome test is used to determine the final sphere. However, as mentioned above, the duochrome test has also been recommended for use before cylinder power correction with the JCC in order to ensure that the COLC is on the retina. This particular use of the duochrome, as explained above, is possibly theoretically unsound, and thus, was investigated in this study. The rationale for this study, then, is the possibly unreliable use of the duochrome before correcting cylinder.

This question was addressed by determining the interval of red-green equality with full corrected cylinder compared to the interval of red-green equality measured without the cylindrical correction, a measure of the utility of the test. Additionally, in those eyes which have only a spherical correction, we induced a cylindrical error, thereby testing the effectiveness of the duochrome under another condition.

## Methods

The research was approved by the Human Studies Committee at Bar Ilan University (Ramat-Gan, Israel), and subjects signed a statement of informed consent prior to their participation in the study. Subjects from the student body and staff at the University volunteered to participate in the study. After autorefractometry and autokeratometry (Topcon KR 7000P, Paramus, NJ, USA) were recorded (RR), the interpupillary distance was measured manually using a millimeter ruler (based on the technique described by Kurtz & Carlson [14]) by RR and verified by LG.

The autorefractometer used in this study works on the Scheiner’s double pinhole principle and a Badal system (which consists of a movable target and a fixed positive power lens located one focal distance away from the eye). Two light sources are imaged in the plane of the pupil to simulate the Scheiner pinhole apertures. A photodetector in the autorefractor observes the degree of coincidence between the two images on the examined retina and automatically adjusts the focus while calculating the refractive power necessary for compensation [16].

It is generally accepted that autorefraction is not sufficiently accurate to provide the optimal spectacle prescription, but can be used as a starting point for the subjective refraction [16]. A study comparing the Topcon autorefractor with subjective refraction found that the two yielded similar spherical equivalent refractive errors, but were significantly different in the astigmatism vector [17]. It should be noted that most autorefractometers provide accurate values for the orientation of the axis and for the power of the cylinder but less so for the power of the final sphere in the correction of the refractive error. In addition, they provide information about the optics of the visual system, but subjective visual acuity and comfort includes higher order processing in the visual cortex and elsewhere, factors that autorefractometers cannot account for. In the present study, the autorefractometer was only used as the starting point for the subjective refraction. Subsequently, all subjects were examined by a licensed optometrist (LG) to determine the full refractive prescription using a Snellen chart with a standard Reichert phoropter, in a three meter room with a mirror creating a distance of six meters, with a full, non-cycloplegic monocular subjective examination and a binocular balance.

### Experimental Protocol

For all subjects, the red-green examination was performed by RR under the supervision of LG. The subject was seated in a moderately darkened room [13, 18] with a rheostat controlled illumination system. The examination was performed monocularly with either spherical correction only or spherico-cylindrical correction. For subjects with a cylindrical correction equal to or greater than 0.75 DC, the test was performed both with and without the physiological cylinder. For subjects with cylindrical corrections lower than or equal to 0.50 DC, a crossed cylindrical lens of +0.50–1.00 x 090 was introduced using the crossed cylinder available in the standard phoropter (similarly to Chen et al. [19]), and the test was performed both with and without this induced cylinder. The order of the eye tested (right or left) and the condition (spherical or sphero-cylindrical) was random. All subjects were fogged by the addition of a +1.00 DS to the prescription placed in the phoropter, in order to ensure that the initial response was under fog, with the red being clearer. The image was projected using a standard screen projector providing high contrast images. Subjects were then instructed to concentrate on the black numbers on the green half of the screen and briefly compare with the black numbers on the red half of the screen and report which background provides a clearer or sharper image. After this response, plus was reduced (or minus added) till equality, followed by additional added minus until entry “into the green”. The prescription yielding the last “red” response, the equality interval, and the first “green” response were recorded.

### Analysis

Data for the right eye only were analyzed to avoid the confounding effect of using non-independent data from both eyes [20]. The data were both analyzed as a group, and separately for the induced cylindrical correction and physiological cylindrical correction sub-groups. The interval between the last “red” response and the first “green” response was calculated for each experimental condition (fully corrected refraction, uncorrected cylinder, and induced cylinder) and compared using a paired, two-tailed Student’s t-test.

## Results

Twenty-seven Bar-Ilan University staff and students (12 male, 15 female) between the ages of 20 and 56 (mean age: 24.56 ± 7.33) participated in the experiment. All were naïve as to the purpose of the experiment. Spectacle wearers were preferred because the goal of the study was to determine if the duochrome test interval of responses differed with differing refractive corrections. As such, the refractive error composition of the recruited subjects is higher than would be expected from the population norms.

Due to the fact that the right and left eyes of the sample are significantly correlated (R = 0.94, p<0.01), and based on Armstrong [21], analysis was conducted on only the 26 right eyes of the subjects (one subject was excluded; see below).

Fifteen of the eyes used in the analysis required a sphere-only correction or a sphero-cylindrical refraction with a cylindrical component less than 0.75 DC. Twelve of the right eyes used in the analysis had a physiological astigmatism larger than or equal to -0.75 DC. See Table 1.

**Table 1:** Subject demographic and optometric details.

Subject #	Age	Gender	OD Rx	OD_SPH INTERVAL	OD_SPH+CYL INTERVAL	DIFF BTW INTERVALS
1	20	F	-1.00/-1.00 X 085	1	0.5	0.5
2	21	F	-4.25/-0.50X065	0.25	0.25	0
3	21	F	-1.75/-1.75X095	0.25	0.5	-0.25
4	21	F	-5.00/-0.75X005	1.25	0.5	0.75
5	21	F	0.25/-0.25 X 180	1	0.75	0.25
6	21	F	-4.25/-0.75 X 060	0.5	0.75	-0.25
7	21	M	-0.25 SPH	0.75	0.5	0.25
8	21	M	-3.75/-0.25 X 095	0.25	0.25	0
9	21	M	-3.50 SPH	0.5	0.5	0
10	22	F	-4.00/-1.50 X 075	0.5	0.5	0
11	22	F	-11.50/-1.25 X 020	0.75	0.5	0.25
12	22	F	-3.25/-1.25 X 165	0.5	0.25	0.25
13	22	F	-3.75/-0.75 X 070	0.75	0.25	0.5
14	22	M	-0.50/-0.50 X 105	0.25	0.25	0
15	22	M	-4.25/ -0.50 X 010	0.25	0.5	-0.25
16	22	M	-5.00/-0.25 X 010	0.5	0.5	0
17	23	F	-0.25 SPH	0.25	0.5	-0.25
18	24	M	-6.00/-0.75 X 125	1	1.75	-0.75
19	25	F	-6.75/-1.00 X 010	1	2	-1
20	26	F	Plano/-0.50 X 030	0.5	0.75	-0.25
21	26	M	-2.00 SPH	1	0.5	0.5
22	26	M	0.50/-2.00 X 090	0.5	0.75	-0.25
23	28	M	Plano/ -0.50 X 100	1	1	0
24	29	F	-3.00/-0.50 X 120	1.25	1.25	0
25	38	M	+3.50/ -2.00 X 090	0.25	0.25	0
26	56	M	-5.00 SPH	0.5	0.5	0

The mean refraction for the eyes used in the analysis of the study was -2.80 ± 3.00 DS (range: -11.50 to +3.50 D) and -0.90 ± 0.55 DC (range: -2.00 to -0.25 D). The mean spherical equivalent refraction of the right eyes was -3.20 ± 3.00 D (range: -12.13 to +2.50 D).

Of the right eyes analyzed in this study, one had simple myopic astigmatism, two had mixed astigmatism, three had simple myopia, five were emmetropes and 16 had compound myopic astigmatism.

**Fig 1:**
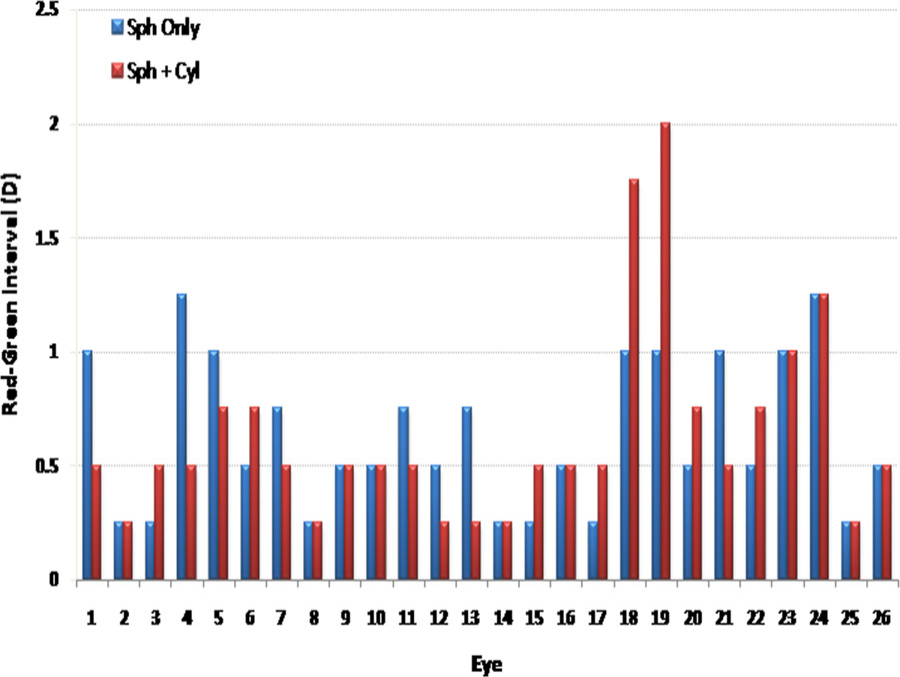
Bar graph of red-green interval. The X axis is subject number and Y axis is the interval between “red” and “green” responses in diopters. Blue is with spherical correction only; red is with spherical and cylindrical correction.

One subject (Age: 20, OD: +0.25 DS simple hyperope, OS: +0.75/-0.25 X 145 compound myopic astigmat, not shown in the table or figure) was not included in the subsequent analysis because she was not able to determine a refraction for which she reported that the “green” was clearer for the sphere-only condition. For the rest of the subjects, the interval between “red” and “green” responses obtained in the sphere-only correction (mean interval: 0.63 ± 0.33 D, range: 0.25–1.25 D) and sphero-cylindrical correction conditions (mean interval: 0.63 ± 0.44 D, range: 0.25–2.00 D) of the right eyes were compared. These intervals, presented in Fig. 1 and tabulated in Table 1, were not significantly different (p = 1.00). The differences between the intervals in the sphere-only correction and sphero-cylindrical correction conditions were between 0 and 0.25 D for 20 subjects, 0.50 D for three subjects, 0.75 D for two subjects, and 1.00 D for one subject. The three subjects for which the interval was 0.50 D were one simple myope, one compound myopic astigmat, and an emmetrope. The two subjects measuring an interval of 0.75 D were a compound myopic astigmat and an emmetrope, and the one subject with an interval of 1.00 D was a compound myopic astigmat with a prescription of -3.00/-0.50 X 120°. Additionally, for eight subjects, the interval was larger for the sphere-only condition, for eight additional subjects, it was larger for the sphero-cylindrical condition, and for ten subjects, there was no difference between the intervals in both conditions. An obvious trend in the refractive error of the subjects with larger intervals was not observed.

The intervals for the subgroup with induced astigmatism and physiological astigmatism with and without cylindrical correction were also not significantly different (p = 0.77 and p = 0.61, respectively). However, these sub-groups were quite small (12 physiological cylinder and 15 induced cylinder) and may have not reached significance due to their size.

## Discussion

The interval of “red” to “green” responses did not differ significantly between the conditions in which the cylinder was corrected and in which it was not corrected. Based on these results it can be concluded that the red-green duochrome test is valid both before the JCC power test and after the JCC test (full cylindrical correction) in the subjective refraction.

As explained, the duochrome test is often used to determine the final spherical correction. However, it is only one of several techniques that can be used as the determiner of the final sphere. In the opinion of many practitioners, it is not the most useful of the various techniques, yet remains popular among many practitioners, especially in Europe. Our results indicate that to whatever degree it is useful, it is effective both prior to cylindrical correction with the JCC and after cylindrical correction has been determined.

One seeming limitation of the study is the underrepresentation of hyperopes in the study sample. However, the prevalence of hyperopia of +3.00 D and higher in adults above the age of 40 in the US, Western Europe, and Australia in the year 2000 was 9.9%, 11.6%, and 5.8%, respectively [22]. In S. Africa, the prevalence of hyperopia of a spherical equivalent value of +1.00 D in adults above the age of 20 is approximately 13% [23]. No study of prevalence of refractive error including hyperopia in Israel currently exists. If it is assumed that the prevalence of hyperopia in Israel is approximately 10% of the population, it would have been expected that a sample of 27 participants would include two or three hyperopes. The study sample included one hyperope and as such, it is possible that our conclusions cannot be generalized for hyperopes. That said, due to the fogging technique used before attaining the COLC position on the retina, resulting in relaxed accommodation, there should theoretically be no difference between myopes and hyperopes on the duochrome test. In addition, the highest cylindrical component of the refraction in the study sample was -2.00 D. As such, it is possible that the results of the study cannot be generalized to patients with high cylindrical refractive components, above -2.00 D.

Another aspect of the study requiring attention is that the accommodation of the subjects was not controlled by means of cycloplegic agents. The experiment simulated natural testing conditions, as would be found in a typical visual examination. The six meter testing distance is assumed to be an infinite distance that does not induce accommodation in emmetropes [24]. As explained above regarding myopes versus hyperopes, undesired accommodation was further avoided in this study by inducing a +1.00 D of fog in front of the eyes prior to conducting the JCC examination. This fog is expected to relax any accommodation that may have been present and is the standard Optometric method used in non-cyclopegicic eyes to achieve this. Therefore, all eyes started out under fog, causing the red side to be clearer. In this situation, accommodation is relaxed. Bringing the eye out of fog by adding minus eventually creates equality of the red and green sides. This results in a controlled process sans accommodation of placing the COLC on the retina and is followed by the use of the JCC for cylindrical correction.

The study sample included young adults and no cycloplegic agent was applied. Often, when examining children a cycloplegic refraction must be performed in addition to the standard subjective refraction. Therefore the results of this study may not necessarily apply to the pediatric population.

Amongst the elderly population confounding factors such as lens opacities which can cause photophobia or myopia exist. Because the study sample consisted of young adults and elderly participants were not part of the present study, the results may not necessarily apply to the elderly population. Similarly, the study population included healthy participants, and the results may not apply to patients with lachrymal film disturbances and dry eye. Finally, the results were not analyzed separately for men and women.

Of the 27 participants in this study, only one (4%) always preferred the red side and never saw the green as clear, and that, only in the spherical-only condition. This type of response is possible in older patients due to lens changes affecting absorption of the short wavelength blue rays, causing the red to appear brighter and more distinct or due to the fact that the chances of a spectacle refraction falling into a 0.25 DS end point are slim.^8^ However, this response was obtained with a 20 year old and was therefore considered idiosyncratic.

Often, symmetrical circular targets (Verhoff circles) are presented during the duochrome test which might enable the eye to average the chromatic aberration in the principle meridians. However, in this study the duochrome target comprised of letters from the Snellen chart presented on a green or red background. Although letter targets can possibly be meridionally biased [25], the results show that this theoretical bias has no effect.

The conclusion reached in this study raises another question, which requires a separate analysis in itself. In the subjective refraction using the JCC for axis and power determination, the JCC test is performed under non-fogged conditions [26], in order to ensure that the circle of least confusion is on the retina. In these non-fogged conditions for power determination as explained above, the intervals of the two JCC presentations cause the COLC to shrink in one case and to expand in the other. This enables quick and reliable addition or subtraction of cylinder power. However, if the JCC procedure is performed under fog, the comparison of the intervals is unreliable. In one presentation, the principal meridians may be closer to each other, but still far enough from the retina to cause significant blur. In the other option, the principal meridians are further apart, but one meridian may be close to the retina without the expected decrease in acuity. Thus, the patient may be misled into erroneously accepting or rejecting cylinder power. Therefore, as is well known, the JCC for power is never performed under fog. However, from our conclusion in this study, where we have shown that the patient perceptually averages out the meridians, the question arises as to what degree of error in refraction would empirically be induced if the JCC for power would be performed under fogged conditions, again contrary to conventional refractive practice.
